# The Diagnostic Performance of Coronary CT Angiography for the Assessment of Coronary Stenosis in Calcified Plaque

**DOI:** 10.1371/journal.pone.0154852

**Published:** 2016-05-05

**Authors:** Liang Qi, Li-Jun Tang, Yi Xu, Xiao-Mei Zhu, Yu-Dong Zhang, Hai-Bin Shi, Rong-Bin Yu

**Affiliations:** 1 Department of Radiology, The First Affiliated Hospital of Nanjing Medical University, Nanjing, PR China; 2 Department of Epidemiology and Biostatistics, School of Public Health, Nanjing Medical University, Nanjing, PR China; University of Washington School of Medicine, UNITED STATES

## Abstract

**Purpose:**

To prospectively evaluate the diagnostic performance of coronary CT angiography (CCTA) for the assessment of coronary stenosis in a calcified plaque, by using conventional coronary angiography (CAG) as a standard reference.

**Materials and Methods:**

Eight hundred and ninety-four patients were known to have or have been suspicious of having coronary artery disease, underwent CCTA and conventional coronary angiography (CAG). All the images acquired were assessed. The calcified plaque in CCTA was classified into four types (I-IV) according to the ratio of calcified plaque volume to vessel circumference (RVTC). Overall diagnostic accuracy was made under receiver operating characteristic curve (AUC) analysis. CAG was used as the standard reference.

**Results:**

A total of 12845 segments were evaluated in 894 patients, among which 4955 calcified plaques were detected on 3645(28.4%) segments by CCTA. The overall AUC, sensitivity, specificity, positive predictive value (PPV), and negative predictive value (NPV) were 0.939, 97.8%, 90.1%, 71.2% and 99.4%, respectively. In type I-II calcification, CCTA had high diagnostic performance in AUC (type I: 0.983; type II: 0.976), sensitivity (96.7%; 98.1%), specificity (99.8%; 97.0%), PPV (95.7%; 90.1%), NPV (99.8%; 99.5%) and accuracy (99.6%; 97.3%). In type III-IV calcification, CCTA has high performance in sensitivity (type III: 97.6%; type IV: 97.9%) and NPV (98.3%; 98.7%), moderate performance in AUC (0.877; 0.829), while remarkable decrease in specificity (78.7%; 67.9%), PPV (71.0%; 56.2%) and accuracy (84.9%; 76.8%).

**Conclusion:**

CCTA has highest accuracy in diagnosing the coronary artery stenosis of type I-II calcified plaques, but has a significant decrease in specificity, PPV and accuracy in type III-IV calcified plaque.

## Introduction

Coronary artery disease (CAD) is the most common cause of death worldwide and thus early diagnosis and timely treatment of this disease can lead to significant reduction in its morbidity and mortality in both younger and older people [1]. Coronary computed tomographic angiography (CCTA) is a well-established invasive imaging modality for noninvasive coronary artery evaluation with high sensitivity and negative predictive value in detecting obstructive coronary artery disease [2–4]. However, the diagnostic performance of CCTA is often hampered by beam-hardening and blooming artifacts due to the presence of highly calcified plaque in the coronary wall. These artifacts can cause an apparent enlargement of the calcified plaque and lead to overestimation or paradoxical underestimation of stenotic severity, that results in high-positive finding on CCTA [5–10], To our knowledge, measuring coronary artery calcium score (CACS) was recognized as a common method to evaluate CAD with severe calcification [11,12]. However, this technique can not accurately and directly measure the stenotic severity of calcified plaque, some studies even illustrate the presence of non-linear relationship between coronary artery calcification and severity of stenosis hence some significant coronary artery stenosis are a result of non-calcified plaques [13–15], thus the CACS validity in evaluating CAD with severe calcification is a controversial issue till date.

In order to improve the diagnostic accuracy for evaluating the stenotic severity of calcified plaque with CCTA, a new method to evaluate its stenotic property is proposed according to the ratio of calcified plaque volume to vessel circumference.

## Materials and Methods

### Study population

From March 2014 to September 2015 we prospectively screened 986 patients who were scheduled for CCTA and conventional coronary angiography (CAG) because they were suspected to have coronary artery disease or progression of a known coronary artery disease was suspected. The exclusion criteria for contrast-enhanced CT included arrhythmia (atrial fibrillation, atrial flutter, or recurrent atrial or ventricular premature beat) (n = 73), renal insufficiency (serum creatinine level >1.5 mg/dL [132.6 μmol/L]) (n = 4), hyperthyroidism (basal thyroid-stimulating hormone < 0.03μL/L) (n = 2), inability to follow breath-hold commands(n = 7), a history of allergic reaction to contrast medium(n = 6).

Thus, a total of 894 patients (526 men and 368 women; mean age, 65.8 ± 6.5 years; age range, 33–82 years) were ultimately included into the study. All patients underwent CCTA and CAG within an interval of 10 days. The local institutional review board of Jiangsu province hospital approved the study and all patients provided written informed consent.

### CT image acquisition and reconstruction

236 of 894 patients (26.40%) were taking β-blockers as a part of their baseline medication. Additional β-blocker medication was not administrated. All patients received a double 1.2mg dose isosorbide mononitrate (Nitroglycerin sublingual).

All CT examinations were performed using a dual source computed tomography scanner (Somatom Definition, Siemens Medical solutions, Forchheim, Germany). Methods applied in the dual source CT study have been described in detail previously [10,16]. In brief, Patients were placed on scanner table in supine position. The scanning parameters for these CT scanners were as follows: a tube voltage set at 100 to 120 kV depending on body mass index, retrospective ECG gating was used, with a gantry rotation time of 330 ms, a tube current of 380–420 mAs, a pitch of 0.2–0.43 (depending on the heart rate), and a collimation width of 64×0.6 mm. Non-ionic contrast medium Iopromide at 370mg/ml (Iopromide 370, Bayer Schering Pharma, Berlin, Germany) was the intravenous contrast medium used in this study. Multiple temporal phase of cardiac cycle was set for ECT-gate retrospective reconstruction. The best diastolic phase and the best systolic phase were selected for evaluation. Effective section thickness of CCTA images was 0.75mm, with a reconstruction increment of 0.4mm. Data sets were filtered with a medium soft tissue convolution kernel (B26f) and a medium-sharp kernel (B46f) were additional used when any calcified plaques were identified. All images were transferred to an external workstation (Syngo Multimodality Workplace Siemens, Siemens, Erlangen, Germany) for further evaluation.

### CT image analysis

For CCTA data analysis, the coronary arteries were assessed by using a 16-segment model according to guidelines of the American Heart Association (AHA) [1]. Small vessel segments (diameter < 1.5mm) and vessel segments distal to occlusions were excluded from analysis. CCTA images were evaluated by using thin-slab maximum intensity projection (MIP), along with curved-planar reformation (CPR) and three dimensional volume rendering (VR).

**Fig 1:**
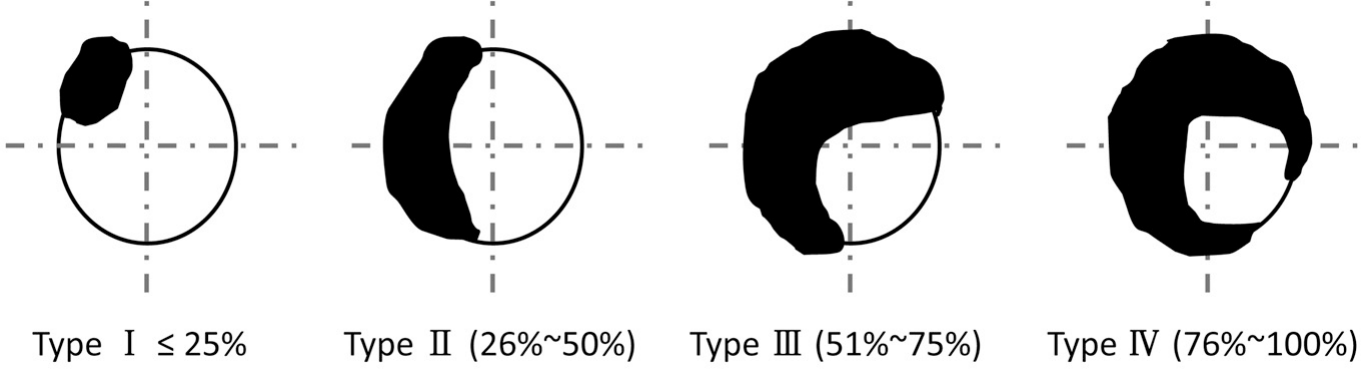
Four types of calcified plaque. Type I: ratio of calcified plaque volume to vessel circumference (RVTC) ≤ 25%; type II: RVTC 26–50%; type III: RVTC 51–75%; and type IV: RVTC 76–100%.

Calcified plaque was defined as plaque composed of >70% calcific volume and was classified into four types according to the ratio of calcified plaque volume to vessel circumference (RVTC), type I: RVTC ≤ 25%; Type II: RVTC 26–50%; type III: RVTC 51–75%; type IV: RVTC 76–100% (Fig 1). This model is a modification of the model devised by Cerci and Vavere AL [17,18].

All images were interpreted by three independent cardiovascular radiologists who had more than 5years of experience and who were blinded to clinical and invasive CAG results. The stenotic severity of calcified plaque was calculated in cardiac image (B46f) automatically by the calculational software. The contour of the ROI was manually corrected. For agreement and repeatability of measurement, three cardiovascular radiologists measured enhanced luminal area in the same calcified plaque six times in total (each one measured twice), and then the mean enhanced luminal area of the six measurements in the calcified plaque were recorded and used for calculation of stenosis.

### Conventional coronary angiography (CAG)

The maximum diameter of stenosis in each calcified plaque was evaluated using quantitative CAG (AXIOM Artis dBA, Siemens Healthcare, Forchheim, Germany). This semi-automatic evaluation was performed by an experienced cardiologist, who was blinded to the CCTA findings. Lesions with a stenosis of 50% or more in diameter were considered to be significant.

### Statistical analysis

All continuous variables were expressed as mean ± standard deviation (SD), while categorical variables were presented as frequencies and percentages. The diagnostic performance of CCTA in evaluating stenotic severity of calcified plaque was presented as sensitivity, specificity, positive predictive value (PPV), and negative predictive value (NPV), accuracy. Receiver operating characteristics (ROC) curve analysis was used to assess the diagnostic performance of CCTA in evaluating severity of stenosis in calcified plaque.

All statistical analyses were performed using SPSS 17.0 (SPSS, Inc, Chicago, IL, USA) and MedCalc version 12.3.0.0 (MedCalc Software, Mariakerke, Belgium).

## Results

Evaluation of diagnostic accuracy was based on a total of 14248 segments among 894 patients. Patient characteristics and calcium score are summarized in Table 1. 119 (0.8%) segments were excluded from analysis because of stent-graft placement; 990 (6.9%) segments were nonaccessible because of the anatomical variants in coronary artery. (n = 54) or small vessel diameter < 1.5mm (n = 936); 231(1.6%) segments were excluded because of motion artifact, 63 (0.4%) segments were excluded because of negative lumen manifestation.

**Table 1:** Patient characteristics.

Characteristics	Value
**Age, years, mean ± SD**	65.8 ± 6.5
**Sex (n)**	
** Men**	526(58.8%)
** Women**	368(41.2%)
**Coronary risk factors (n)**	
** Hypertension**	777 (86.9%)
** Diabetes**	196 (21.9%)
** Smoking**	585 (65.4%)
** Dyslipidemia**	604 (67.6%)
** Family history CAD**	257 (28.7%)
**CCTA HR, beasts/min**	
** Mean ± SD**	64.6 ± 5.3
**Agatston Calcium Score**	312.6 ± 548.7

SD, Standard Deviation; CAD, coronary artery disease; CCTA, coronary computed tomography angiography; HR, heart rate.

**Fig 2:**
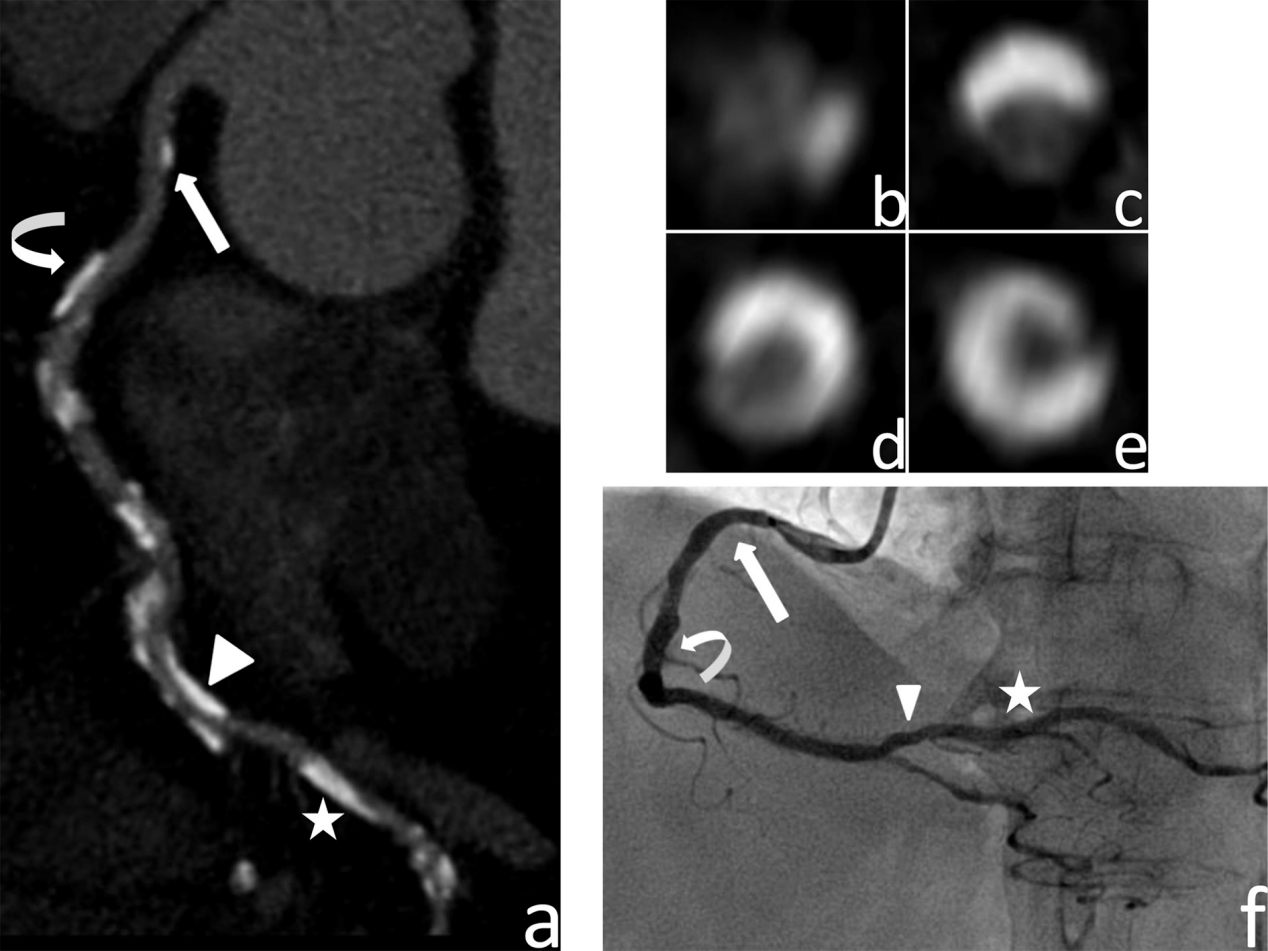
A 67-year old male presenting with typical chest pain for about 2 months. (a) Curved multiplanar reconstruction, (b, c, d, e) axial images and (f) conventional coronal angiography of right coronary artery in a 68-year-old patient. The calcified plaque (arrow) in segment 1 was classified as type I according to its corresponding axial image (b), the stenosis severity of type I calcified plaque was about 8% that was evaluated by CCTA (arrow), and CAG shows no significant stenosis (arrow); Another calcified plaque (curved arrow) in segment 1 was classified as type II according to its corresponding axial image (c), the stenotic severity of type II calcified plaque was about 37% evaluated by CCTA (curved arrow), and the CAG shows no significant stenosis (curved arrow); The calcified plaque (arrow head) in segment 3 was classified as type III according to its corresponding axial image (d), the stenotic severity of type III calcified plaque was about 68% evaluated by CCTA (arrow head), while the CAG shows mild significant stenosis (< 50%) (arrow head); Another calcified plaque (asterisk) in segment 3 was classified as type IV according to its corresponding axial image (e), the stenotic severity of type IV calcified plaque was about 82% evaluated by CCTA (asterisk), while the CAG show no significant stenosis (asterisk).

In remaining 12845 segments, 4955 calcified plaques were detected on 3645(28.4%) segments by CCTA, of which 1769 (35.7%) were Type I, 1248 (25.2%) type II, 947 (19.1%) Type III and 991 (20.0%) Type IV calcified plaque (Fig 2).

**Fig 3:**
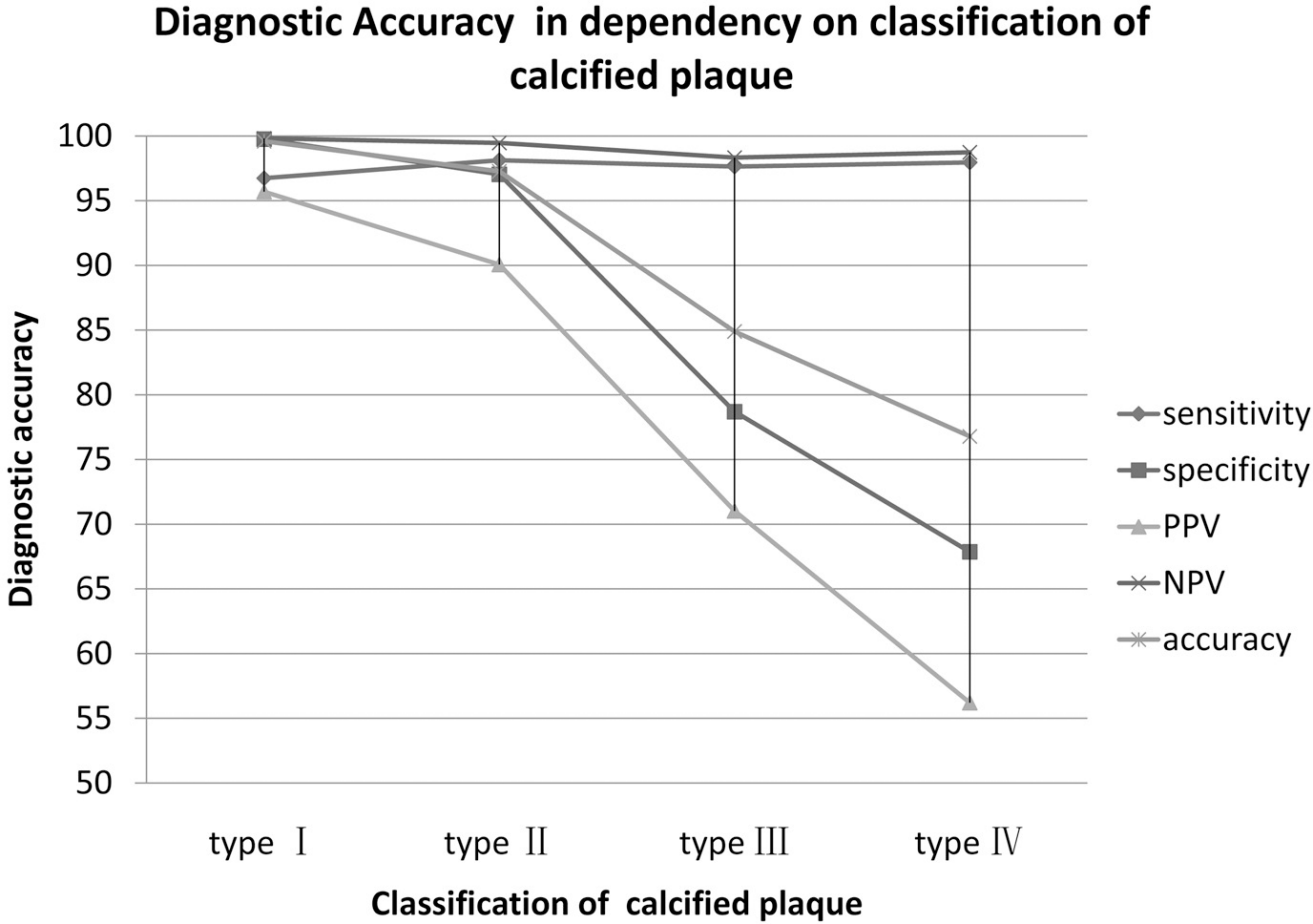
A chart of diagnostic accuracy of CCTA for evaluating severity of stenosis in different type of calcified plaque. Sensitivity and NPV showed a minor variation in type I to IV calcified plaques. While specificity decreased from 99.8% (type I) to 67.9% (type IV), PPV decreased from 95.7% (type I) to 56.2% (type IV), and accuracy decreased from 99.6% (type II) to 76.8% (type IV).

Evaluation of stenotic severity in calcified plaque using CCTA was compared to the standard reference of CAG. In 1769 type I calcified plaque which were manifested in CCTA, 1762 (99.6%) calcified plaques were correctly diagnosed, 3 (0.2%) calcified plaques were misdiagnosed and 4 (0.2%) calcified plaques were overestimated in CCTA. In 1248 Type II calcified plaques, 1214 (97.3%) calcified plaques were correctly diagnosed, 5 (0.5%) calcified plaques were misdiagnosed and 29 (2.2%) calcified plaques were overestimated in CCTA. In 947 Type III calcified plaques, 804 (84.9%) calcified plaques were correctly diagnosed, 8 (0.8%) calcified plaques were misdiagnosed and 135 (14.3%) calcified plaques were overestimated in CCTA. In 991 type IV calcified plaques, 761 (76.8%) calcified plaques were correctly diagnosed, 6 (0.6%) calcified plaques were misdiagnosed and 224 (22.6%) calcified plaques were overestimated in CCTA. According to the results mentioned above, the sensitivity, specificity PPV, NPV and accuracy is shown in Table 2. CCTA shows the high accuracy (91.6%) for all calcified plaques, the sensitivity (97.8%) and NPV (99.4%) is excellent, the specificity (90.1%) is relatively high, while the PPV (71.2%) is moderate. Sensitivity showed a minor variation with values between 96.7% (type I) and 98.1% (type II). Similarly, NPV showed a minor variation between 99.8% (type I) and 98.3% (type III), while specificity decreased from 99.8% (type I) to 67.9% (type IV), PPV decreased from 95.7% (type I) to 56.2% (type IV), and accuracy decreased from 99.6% (type II) to 76.8% (type IV) (Fig 3).

**Table 2:** Diagnostic accuracy of CCTA for evaluating stenosis severity of calcified plaque.

Strata	NCP(n)	TP (n)	TN (n)	FP (n)	FN (n)	SEN (%)	SPE (%)	PPV (%)	NPV (%)	Accuracy (%)
**All**	4955	971	3570	392	22	97.8	90.1	71.2	99.4	91.6
**Type I**	1769	89	1673	4	3	96.7	99.8	95.7	99.8	99.6
**Type II**	1248	263	951	29	5	98.1	97.0	90.1	99.5	97.3
**Type III**	947	331	473	135	8	97.6	78.7	71.0	98.3	84.9
**Type IV**	991	288	473	224	6	98.0	67.9	56.2	98.7	76.8

NCP, number of calcified plaque; TP, true positive; TN, true negative; FP, false positive; FN, false negative; SEN, sensitivity; SPE, specificity; PPV, positive predictive value; NPV, negative predictive value.

**Fig 4:**
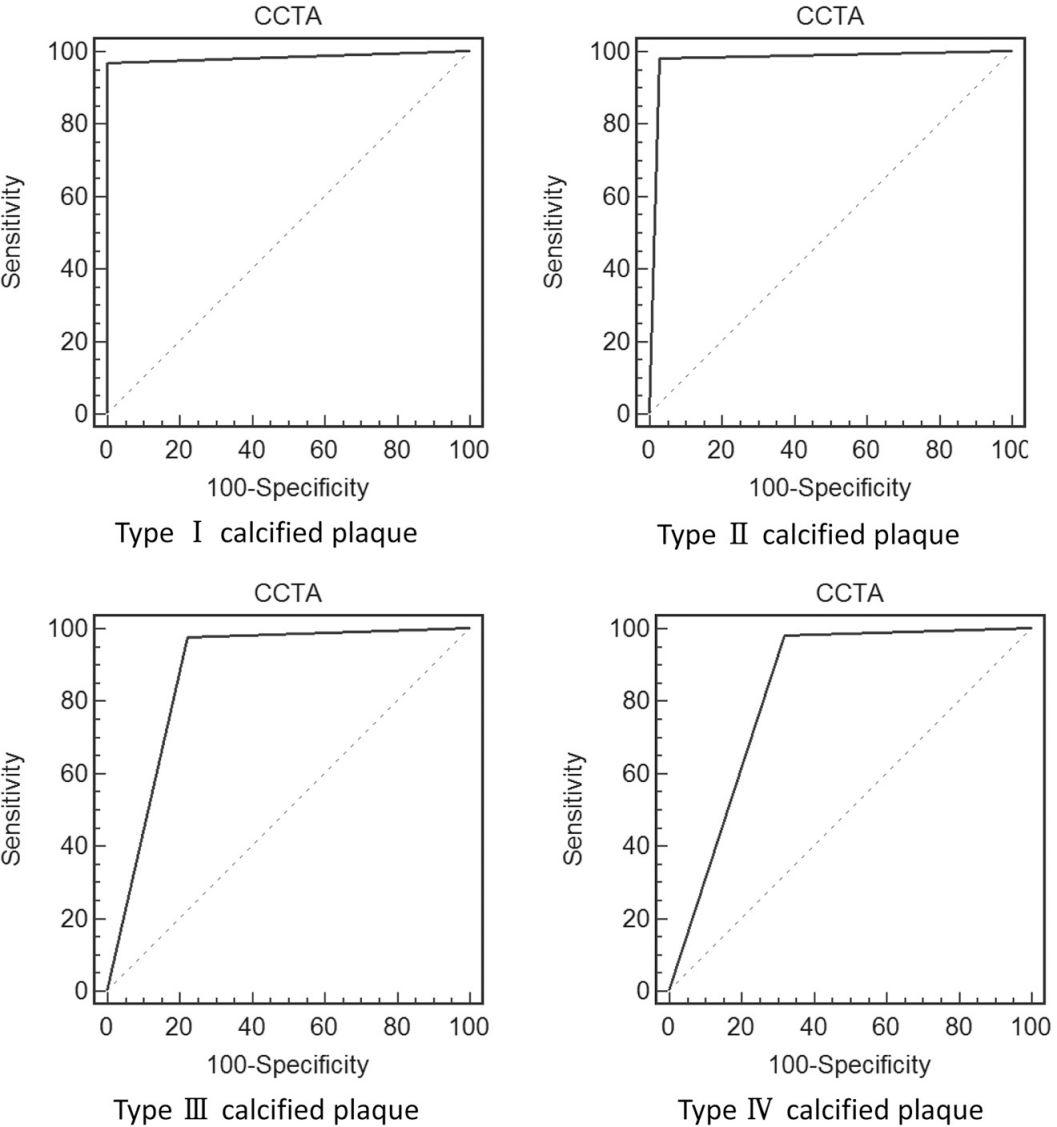
Receive-operating characteristics (ROC) curves illustrate the CCTA ability of evaluating significant stenosis due to different types of calcified plaque. The areas under the ROC curves (AUCs) are 0.983, 0.976, 0.877 and 0.829 respectively for type I to IV calcified plaques.

As determined via receiver operating characteristic curve (ROC) analysis, the area under the ROC (AUC) was 0.939 in all calcified plaques. The AUC was found to be 0.983, 0.976, 0.877 and 0.829 respectively in type I to IV calcified plaques (Fig 4), the AUC was relatively high for type I and II calcified plaque, and was significantly decreased in type III and IV calcified plaque.

## Discussion

CCTA is an accurate method for noninvasive diagnosis or exclusion of coronary artery disease. However, blooming artifacts caused by calcified plaque have been recognized as the essential hardship in accurate detection of vessel stenosis [5–9]. In order to improve diagnostic performance in CCTA for calcified lesions, within this study, we tried to classify the calcified plaques into four types according to the ratio of calcified plaque volume to vessel circumference. In groups of type I and II calcified plaques, CCTA showed a high diagnostic accuracy. However, in groups of type III and IV calcified plaque with high sensitivity and NPV, CCTA only showed a moderate specificity (78.7%), PPV (71.0%) and accuracy (84.9%) in type III calcified plaques, and low specificity (67.9%), PPV (56.2%) and accuracy (76.8%) in type IV calcified plaques, reflecting its limitation in stratifying vessel stenosis with type III and IV calcified plaques.

In our study, 4955 Calcified plaques were detected on 3645 (28.4%) segments by CCTA. In order to reduce blooming artifacts of calcified plaque, the severity of stenosis in calcified plaque were calculated in CCTA images which were reconstructed using a conventional FBP (filtered back-projection) algorithm with an edge-enhancing tissue convolution kernel (B46f), this method was used previously in literature [17,19,20]. The overall sensitivity, specificity, PPV, NPV and accuracy were 97.78%, 90.11%,71.23%, 99.39% and 91.64%, respectively, which is partly compatible with the previous literature studies [16,21–23]. However, the specificity and PPV in our study remains relatively negotiable than the previous literature studies [21–26]. This may be due to the following reasons. Firstly, in our study, only the calcified plaque in segments were prospectively involved. The blooming artifacts caused by the calcified plaque have high influence on the image quality and thus reduce the accuracy of performance; Secondly, CCTA images reconstructed previously used medium-smooth kernel instead of an edge-enhancing tissue convolution kernel for imaging of severely calcified coronary arteries, which may affect the diagnostic accuracy for evaluating severity of stenosis in calcified plaque with CCTA; Thirdly, in our study, we focused only on the analysis of calcific plaque that had been composed of >70% calcific volume, which mainly had an impact for the diagnosis of CAD, while other previous studies may have included a variety of calcified plaques such as noncalcified, partially calcified, or calcified plaque.

In our collected data set, a total of 1769 (35.7%) type I calcified plaques were detected on CCTA. The sensitivity, specificity, PPV, NPV and accuracy was performed well and the results were excellently compatible with previous studies. Hence it indicates that the type I calcified plaque rarely affect the evaluation of coronary stenosis by CCTA. In 1248 (25.1%) type II calcified plaques, the sensitivity, NPV and accuracy were generally well performed, although the specificity and PPV slightly decreased when compared with type I calcified plaque. It suggests that the type II calcification also slightly affect the imaging performance as in type I. In 947(19.1%) type III and 991 (20.0%) type IV calcified plaques, CCTA only showed a good sensitivity and NPV, however, the specificity, PPV and AUC were significantly reduced, when compared to the results of type I and type II calcified plaques. It indicates that the blooming artifacts from type III-IV calcified plaques significantly affects the evaluation of severity in coronary artery stenosis on CCTA. These results from our study are compatible with those of previously similar literature studies [17,18,24–28]. The series by Palumbo et al, Park et al, Meng et al and Chen et al reported that the calcification of coronary arteries does affect the diagnostic accuracy of CCTA, with the increase of calcification in coronary artery it significantly decreases the diagnositc accuracy. The studies by Cerci et al, Kruk et al and Vavere et al also indicated that the calcium characteristics enhances the inaccuracy of CCTA in lumen assessment within calcified lesions. The diagnostic accuracy decreases while the coronary atery cross section calcium area increases. In Cerci’s study, it demonstrated that only 4% of ≥50% stenoses by quantitative coronary angiography were very severely calcified, most ≥50% coronary artery stenoses are not or only mildly calcified, it means that the lesion with most severe calcification is often not the one with the highest degree of stenosis.

Our study has some limitations. Firstly, this is a single-central study, the results from it represent a prospective experience involving 4955 calcified plaques and thus requires confirmation involving larger multi-central studies. Secondly, the calcified plaque was defined as plaque composed of >70% calcified volume, thus, the results of our study might not apply to partially calcified plaque (<70%). Thirdly, in some cases, the density of the luminal enhanced area and calcified plaque are very much similar to each other, and are hard to be distinguished. Fourthly, we only focused on analysis of the calcified plaque with composition of >70% calcified volume whilst excluding other types of plaques as that have been illustrated previously in the literature [10,17,28].

## Conclusion

Within this study, we prospectively evaluated the diagnostic performance of CCTA for the assessment of coronary artery stenosis with calcified plaques. CCTA showed excellent sensitivity and NPV in diagnosis of coronary stenosis for all-types of calcified plaques, and showed excellent specificity and PPV in type I-II calcified plaques. However, as a result of blooming artifacts caused by calcification, CCTA showed a moderate to low specificity and PPV in type III-IV calcified plaques. Thus for type III-IV calcified plaques in CCTA, it may have to be to combined with CACS for accuracy and if necessary CAG should be considered.

## Supporting Information

S1 Table Data of The diagnostic performance of coronary CT angiography for the assessment of coronary stenosis in calcified plaque. (XLSX)
